# An exploration of sensory and movement differences from the perspective of individuals with autism

**DOI:** 10.3389/fnint.2012.00107

**Published:** 2012-11-16

**Authors:** Jodi Robledo, Anne M. Donnellan, Karen Strandt-Conroy

**Affiliations:** ^1^School of Education, California State University San MarcosSan Marcos, CA, USA; ^2^School of Leadership and Education Sciences, University of San DiegoSan Diego, CA, USA; ^3^Parkview School DistrictOrfordville, WI, USA

**Keywords:** autism, sensory and movement differences, first-person accounts

## Abstract

Parents, teachers, and people who themselves experience sensory and movement differences have consistently reported disturbances of sensation and movement associated with autism. Our review of the literature has revealed both historical and recent references to and research about sensory and movement difference characteristics and symptoms for individuals with autism. What is notably infrequent in this literature, however, is research that highlights the perspective of the individual with autism. If we wish to truly understand the experience of sensory and movement differences for individuals with autism, we must explore their experiences and perspectives. This study presents a qualitative analysis of more than 40 h in-depth inquiry into the lives of five individuals with the autism label. Data were sorted into six categories: perception, action, posture, emotion, communication, and cognition. The insights into sensory and movement differences and autism offered by these individuals was illuminating. We found that the data strongly supported the presence of disruption of organization and regulation of sensory and movement differences in the lived experience of these participants with autism. The present data suggests that in autism this disruption of organization and regulation is amplified in terms of quantity, quality, intensity, and may affect everyday life. These data contribute to a more expansive view of autism that incorporates the possibility that autism is a disorder that affects motor planning, behavior, communication, the sensory motor system, and the dynamic interaction of all of these.

## Introduction

The history of autism reflects the prevailing understandings and misunderstandings about human development and communication that characterized professional writings in psychology, psychiatry, and special education over the past 60 years. In the absence of a clear understanding of cause or symptoms, many definitions and theories about autism have been developed. Most often the descriptions offered by the professionals pay little attention to the experience of people who live with autism.

Seventy years after Leo Kanner's original paper on autism (1943), the orthodox “scientific” thinking is that autism is a separate psychiatric disorder, reliably distinguishable from other human conditions, likely the result of absence or error in or affecting the social brain. Moreover, the abilities of the person with autism can be reliably and validly determined through our psychological and behavioral assessments (e.g., *DSM-IV-TR*, American Psychiatric Association, 2000). This view draws on a dualistic tradition in psychology and psychiatry that separates mind and body (seeRogers, 1990; Damasio, 1994). It leaves out a long and rich history of writing and research which suggests that individuals with a variety of disabilities or disorders may, in fact, be experiencing differences in their sensory, motor, perceptual, and other systems, which confound and confuse the picture (e.g., Kahlbaum, 1874/1973; Bleuler, 1911/1950). A series of papers by Donnellan, Leary, and Hill spells out in detail the effect this dichotomy has had on our understanding of autism (Hill and Leary, 1993; Donnellan and Leary, 1995; Leary and Hill, 1996; Leary and Donnellan, 2012). They posit that assuming mind can be studied separately from body ignores the importance of felt experience on the development of social interaction, communication, and behavior. Even in the more recent research that studies the body (motor differences) and autism, there is little understanding of the potential affect of these differences on social, communication, and behavioral functioning (see Leary and Donnellan, 2012,^1^).

Leary et al. (1999, as cited in Donnellan et al., 2006) have defined a sensory and movement difference as a difference, interference or shift in the efficient, effective utilization and integration of movement; a disruption in the organization and regulation of perception, action, posture, language, speech, thought, emotion, and/or memory. Typically, the word “movement” refers to observable actions, such as posture, muscle tone, head and eye movements, facial expression, vocalization, speech, whole body movements, reaching, gesturing, running, and walking. Here, the use of the word movement is consistent with research that considers internal mental processes of sensory perceptions (touch, taste, smell, vision, hearing, and proprioception), language, thoughts, and emotions as aspects of human movement.

A review of published first-hand accounts of autism and research studies with participants with autism revealed numerous references to sensory and movement differences in the areas of perception, action, emotion, communication, and cognition. Each area will be briefly described below.

Perceptual differences, such as differences in hearing, vision, smell, taste, proprioception, and synesthesia were all noted in numerous published first-hand accounts (e.g., White and White, 1987; Cesaroni, 1990; Barron and Barron, 1992; Grandin, 1992, 1995; Williams, 1992, 1994; McKean, 1994; Blackman, 1999; Mukhopadhyay, 2000; Rubin, in Biklen, 2005). Tito Mukhopadhyay described his perceptual experiences as a *“fragmented world perceived through isolated sense organs”* (2000, p. 74). Jim, a research participant in a study conducted by Cesaroni indicated, *“Sometimes I know that something is coming in somewhere, but I can't tell right away what sense it's coming through”* (Cesaroni, 1990, p. 74).

First-hand accounts of autism also revealed challenges with controlling, executing, and combining action or movements (Volkmar and Cohen, 1985; Cesaroni, 1990; Grandin, 1992; Williams, 1992, 1994; McKean, 1994; Hale and Hale, 1999; Mukhopadhyay, 2000; Frugone, in Biklen, 2005; Mukhopadhyay, in Biklen, 2005; Goddard and Goddard, 2012). Alberto Frugone described his challenges with action and movements: *“Right from the beginning of an action, I was conscious of my inability to access motor planning and I was lost in an unacceptable motor silence”* (Frugone, in Biklen, 2005, p. 190). Charles Hale described his difficulty with actions and movements:

I think my movement disorder is most apparent in the fact that I am unable to respond to someone or something, when my intelligence would tell me to respond in an appropriate manner. For instance, when I should be smiling, sometimes I know that I am not smiling but may be even frowning. This causes me a great deal of pain and makes me look as though I am not comprehending when, in fact, I am crying to respond in an appropriate manner. (Hale and Hale, 1999, p.32)

First-hand accounts of autism described challenges with regulating emotions (e.g., Cesaroni, 1990; Barron and Barron, 1992; Jolliffe et al., 1992; Williams, 1992, 1994). Sean Barron described that he could not control his emotions and he was terrified of his “feelings and temperament” (Barron and Barron, 1992, p. 118). Many first-hand accounts described stressful feelings and anxiety as predominant emotions. Another individual with autism, Therese Jolliffe commented: *“It [stress] occurs at any time, but always when I know I have to go somewhere stressful. Sometimes the pain is so bad that my whole body becomes stiff and then I am unable to move.”* (Jolliffe et al., 1992, p. 14)

Communication challenges were also noted in numerous first-hand accounts of autism (e.g., Cesaroni, 1990; Barron and Barron, 1992; Jolliffe et al., 1992; Williams, 1992, 1994; Grandin, 1995; Blackman, 1999; Mukhopadhyay, 2000; Rubin, in Biklen, 2005; Goddard and Goddard, 2012). Sue Rubin, a non-verbal individual with autism who independently uses augmentative and alternative communication described her difficulty with initiating speech: *“I rarely find the strength in my autistic capabilities to initiate a conversation. There may be times where something pertinent eats away at me until either I find a moment where my body and mind coincide and I am able to go get a device to converse with.”* (Rubin, in Biklen, 2005, p. 85)

Individuals with autism in first-hand accounts also described differences in cognition (e.g., Jolliffe et al., 1992; Williams, 1994; Grandin, 1995). Temple Grandin (1995) outlined her thought process in her book entitled “*Thinking in Pictures*.” She explained that she translates spoken and written words into *“full-color movies, complete with sound, which run like a VCR tape”* (p. 19) in her head. She labeled this technique as visual thinking. To create new images, she takes parts of *“video memories”* (p. 21). To recall a memory she replays various video memory tapes until she finds the information she is searching for. Her videos, however, sometimes trigger a series of free associations. Sometimes certain words can also trigger the incorrect association and she can look for an incorrect video, which she says leads to misunderstandings.

It is essential that the exploration of autism include sensory and movement differences and involve the people who experience autism first-hand for a number of reasons: (1) professionals investigating autism from a perspective that separates mind and body may have overlooked sensory and movement differences, and/or their possible effect on behavior; (2) published first-hand accounts of autism suggest that this is a fruitful area for investigation; (3) in studying autism we need to elicit information from one of the most valuable resources—people with the label of autism.

Most of the disciplines studying autism have investigated autism through clinical research looking at significant group differences. This pursuit has brought valuable information but, in addition, has brought about confusing, confounding, and contradictory results. Researchers are beginning to explore the experience of autism through a critical disability perspective by including the perspective and experience of people with autism (e.g., Strandt-Conroy, 1999; Broderick and Kasa-Hendrickson, 2001; Biklen, 2005; Robledo and Donnellan, 2008). Biklen (2005) summarized the importance of qualitative methodologies in a field that has been dominated by positivist research:

In one central way, their accounts diverge dramatically from the prevailing clinical literature … Their richness suggests the danger of privileging other forms of research about autism as more deserving of authority or as being in some way uncontestable. Their forcefulness and consistency should signal clinical researchers to question every assumption brought to the topic of autism. (p. 281)

## Materials and methods

A qualitative research design was utilized in this study to gather data aimed at describing the experience of sensory and movement differences in individuals with autism. Qualitative designs foster interpretations and descriptions that allow understanding of the concept being studied (Ferguson et al., 1992). Further, qualitative research attempts to create a naturalistic paradigm in which the researcher is able to understand an individual's experiences.

### Participants

Five people with the diagnosis of autism participated in this study. It was anticipated that obtaining data from this population would be challenging due to the well-documented social, communication, and behavioral challenges experienced by many people with this diagnosis. For that reason, the researchers used a variety of methods to obtain meaningful data. These methods included in-depth interviews, questionnaires, and participant observations. All aspects of this study conformed to the Institutional Review procedures of the School of Education, University of Wisconsin-Madison.

There were five-independently communicating participants in this study. Each person was sought through a number of associations involving people with autism. They were each contacted regarding the research and depending on their personal preferences, were provided with background information regarding their involvement. Subsequent to this, informed consent was sought. At the outset of the study, participants were asked to provide information regarding his or her autism, including Asperger's syndrome or autism spectrum disorder. Confirmation of diagnosis was obtained from a review of past records, interviews with family or involved staff members, and/or direct observation of the individual. Although we intended to use pseudonyms, all participants asked that their real names be used. For purposes of this paper, only the participant's first names were used. A brief description of each participant at the time of data collection follows.

Geneva was a 57-years-old female. At age six, Geneva was diagnosed with encephalitis. Early in Geneva's educational experience she received little to no help in school despite the fact that, she was having difficulties. In third grade, school staff investigated Geneva's learning problems. Testing was done but no assistance was given to her. It was not until junior college that the discrepancy between Geneva's receptive and expressive language skills was discovered. She was tested and given a list of her learning disabilities. Subsequent diagnostic labels include: Aphasia, Dyslexia, Sequential Learning Deficit, and Epstein Barr Syndrome. Later in life she went to doctors including Dr. B. J. Freeman at the University of California Los Angeles's Neuropsychiatric Institute, and was diagnosed with a form of high functioning autism known as Asperger's syndrome.

Jean Paul was a 29-years-old male. He was diagnosed with autism and mental retardation at age 3. Professionals recommended to his mother that he be placed in an institution while she underwent psychotherapy. During his early days in school, an attempt was made to place Jean Paul in a program designed for the cognitively disabled. Jean Paul's mother did not listen to the medical or school personal. Instead she worked very hard to provide her son with the needed resources. As a result of Jean Paul's and his mother's hard work, Jean Paul has two college degrees.

Kathy-Xania was a 33-years-old female. Kathy-Xania remembered experiencing information and her surroundings differently from an early age. In her early childhood, her father, a physician, sought out advice but disregarded many of the explanations of autism given to him during the late 1960s and early 1970s. Kathy-Xania was appreciative of that fact and said that as a result of her father's actions, she was educated with her non-disabled peers. Kathy-Xania attended college and earned her bachelor's degree. She is divorced from her husband and lives independently.

Barbara was a 40-years-old female. At an early age Barbara was placed in a psychiatric institution. At age 17 she became an outpatient of the institution and lived with a foster family for 10 years. During that time she attended 1 year of college. She decided to leave college and began working in the kitchen of a nursing home. She held the job for 22 years despite her dislike of the job. She lives independently receiving support from her siblings, especially her sister, Ruth.

Matt was a 19-years-old male. Matt's parents were given a diagnosis of autism when Matt was 18 months old. His mother was very supportive of Matt and advocated for his inclusion in school and the community. Matt received special education support throughout his school years. During high school, Matt attended all regular education classes. Matt is extremely skilled in mathematics and subsequent to his participation in this study he received a bachelor's degree in mathematics from a major university.

### Instrumentation and data collection

Each participant was asked to identify their preferred method(s) for data collection (i.e., face-to-face or phone interviews, completion of written questionnaire, and/or participant observation). In order to optimize their participation in the interview process, those interested in completing interviews were given a choice as to how, where and when they would like to be interviewed. Additionally, any accommodations an individual needed to make the interview more productive were provided. One such example included the option to respond to oral questions in writing.

Depending on individual preference and the extent to which individuals were able to participate in extended interviews, multiple interviews of varying length and format were completed. While no set number of interviews was required of participants, the total time taken to complete interviews was in the vicinity of 40-h. Prior to beginning each interview, the researcher conducting the interview talked informally with the participants to create rapport.

If the participant wished to complete a questionnaire packet, he/she was asked to select a method of sharing information (e.g., E-mail, U.S. Mail or telephone). Also, any accommodations needed to make the questionnaire more productive were provided and included the option for further oral explanation of questionnaires or the completion of portions of the questionnaire during telephone conversations.

In addition to the use of interviews and questionnaires, participant-produced data (e.g., drawings, writings) were reviewed and observations of four of the five participants who selected face-to-face interviews were completed.

The researchers approached the interviews from a primary principle of qualitative interviewing by providing “a framework within which respondents can express their own understandings in their own terms” (Patton, 2002, p. 205). In order to provide a framework, a minimally structured interview format was used.

A variety of predetermined questions were derived from the professional literature and published first-hand accounts of autism. These questions were used as a guide rather than a script during the interview. Possible interview questions were sorted into nine categories: hearing, vision, touch, action, posture, emotion, communication, cognition, and general. Examples of interview questions included requests for information on the extent to which elements in the environment provoked an adverse response in the individual, whether movement or control of the body was problematic, and what accommodations assisted the individual in these situations. Upon completion of questions in a specific topic area, participants were asked if they had any additional information or examples they wished to add. Information provided by the participants was reviewed after each interview and was used to develop structured questions for subsequent interviews.

Four of the five participants stated that the phone interview was a better format because it allowed them to be more active in the dialog. One participant described that they were best able to respond to questions during a phone interview if they were also taking a bath. To ensure adequate information from the participants they were asked to think about certain topics ahead of time and additional response time was offered.

A questionnaire written at approximately the 6th grade level was created using the professional literature and published first-hand accounts of autism. Vignettes incorporating the themes introduced earlier were presented in the questionnaire packet and participants were asked about the extent to which their own experiences were similar to or different from those in the vignettes. After each vignette additional questions were asked. Questionnaires included short answers, multiple choice, and fill-in the blank answers.

Along with the audiotaping of each of the interviews, memos and field notes during and after each interview were completed (Lincoln and Guba, 1985). Memos involved the researchers in writing ideas, thoughts, assumptions, concepts, and relationships between concepts that emerged while interviewing, coding the data, consulting with others, or contemplating what had occurred during data collection (Strauss, 1987).

The researchers reviewed portions of dairies, newspaper articles, drawings, poems, and photographs from three of the five participants. Any artifact or documentation (i.e., diaries, drawings) that the participant wished to share with the researchers was used for document and artifact review. Memos were written up about each document. These artifacts assisted the researchers with gaining an in-depth and more complete understanding of the participant.

### Data analysis

Data analysis occurred throughout the data collection phase using the constant comparative approach (Charmaz, 2000, 2006). This allowed the researchers to collect data, analyze it, and then collect additional data. Information was shared with participants to confirm interpretations of the data. As the participant data was collected, all the interview transcripts were analyzed and themes or categories were changed or expanded upon. For example, the themes “memory” and “thoughts” were combined into the category of “cognition.” Initially, data were coded using two descriptors that were typed before each excerpt. The first descriptor was the area (e.g., perception/vision). The second descriptor was a specific attribute (e.g., difficulty integrating visual stimuli). After these two descriptors had been assigned, data were sorted into categories. These excerpts were again read and changes were made to the categories and codes. After all the data were collected from the participants, all interview transcripts, questionnaires, coded data, and researcher notes including notes from the document review, and notes from listening to the audio tapes were re-analyzed to confirm themes/categories and codes.

## Results

For clarity of presentation data will be presented in six categories: perception, action, posture, emotion, communication, and cognition. It is important for the reader to note that there is a dynamic interaction among these areas as no category operates in isolation.

### Perception

Sensory and perceptual differences related to hearing, vision, and touch were common in the experiences of participants. Challenges with proprioception were also described; however, these findings will be presented in the “posture” category. Some participants indicated differences in smell and taste, but they will not be reported here as differences in hearing, vision, and touch yielded the greatest amount of data.

#### Hearing

Auditory differences were noted in the experiences of participants. For some, certain sounds evoked physical pain and anxiety. For others, sounds elicited emotions not necessarily related to the present context. Some participants indicated differences in their ability to selectively focus on auditory input.

Jean Paul indicated that ambulances, airplanes taking off, and loud screechy noises are problematic, creating a sense of *“having the jitters.”* Similarly, Matt explained his painful reaction to certain sounds:

Especially things like gunshots, loud motors, and brass bands. My mom took me through a drive-thru car wash once when I was in grade school and I was terrified. The brushes sounded to me like the sound of intense machine gun fire, but I could not communicate well enough to explain why I got so upset.

Barbara discussed how sounds triggered her emotions. She said, *“It seems like there is something in my brain that certain noises trigger my emotions the way pain does.”* She described how the sound of a crying baby could *“agitate and anger”* her. Barbara further explained:

Some sounds make me feel really bad in the pit of my stomach. I feel angry and aggressive and out of control; feeling aggressive towards someone who doesn't deserve it makes me feel guilty. I get very agitated. I may yell at people. My behavior gets out of control. It can ruin my mood sometimes for days. The effects of the noise last much longer than the noise itself.

She expressed concerns during a phone interview as the researcher conducting the interview had recently had a baby. Barbara was very fearful that she would hear the baby over the phone. She explained, “*If you really understand how I feel about babies, you'd move heaven and earth to keep that baby away from me.”*

Through her questionnaire she indicated that these emotional reactions caused her problems including:

a) I complain a lot and then get criticized a lot; b) If I know I can't get away from bad sounds I get irritated and depressed; c) If I anticipate a situation where there may be bad sounds, I get depressed, feel helpless; d) sometimes a loud sound will provoke me to tears.

Whenever Barbara is in public she wears earplugs and a radio headset. She said, *“this can provide competing sound which may be distracting so I won't focus so much on the bad sounds.”* She also indicated that she was appreciative for those who allow her to leave noisy situations: *“It really helps when other people understand. I feel guilty mostly because of others' reactions. It's painful to be criticized.”*

Kathy-Xania noted that she experienced times when sounds faded in and out making it difficult at times to focus on auditory input. She hypothesized that when there was too much *“static”* in the sound it was more likely to fade in and out. Kathy-Xania, noted:

It is hard to hear when a person has static in their voice. I don't like it when babies cry. I don't like static. I don't like high-pitched noise. I don't like hearing gunshots. I do hear gunshots. I don't like gunshots, I don't like kids screaming. I don't like staticky voices. I don't like some of those old women who have those horrible voices.

#### Vision

Participants described several different types of visual differences: unique interactions with colors, stimulation or pain caused by visual stimuli, different responses to lighting, and challenges with eye contact.

Primarily, participants spoke about negative reactions to visual stimuli. Barbara, however, described that at times she greatly enjoyed bright lights and certain combination of colors. For example she described enjoying looking at traffic lights, *“if they are put together right; modern ones disappoint me.”* Barbara also indicated she had a strong need for visual stimulation:

In other words, I crave light and colors. I always feel my best on a bright, sunny day. I like rooms to be brightly lit and if you saw my apartment, you'd see that I papered the walls with all kinds of pictures. I turned an art gallery out of it.

Kathy-Xania also spoke about her need for bright light. She indicated that she has been told that she experiences seasonal-affective disorder. She described that a few days of cloudy weather affects her adversely, leaving her feeling sad and depressed. She explained, *“I can feel my body chemistry change when there is sun.”*

Jean Paul, Matt, and Geneva spoke about negative and painful reactions to certain visual stimuli. Geneva said, *“There are certain types of light I cannot tolerate—they make me nervous. If I am in a hall and it is too bright, I can't handle it, I have to put a sun hat on.”* She also said she was not able to handle fluttering fluorescent lights because that type of light *“absolutely turns my stomach into knots. It does a trip on my nervous system.*” Even the sun can be problematic. Geneva explained that when she walks out the door on a bright day her eyes take up to 3 min to adjust:

Because it hits my head like a lightening bolt. I have to stand there with my eyes closed and hold on to something so I don't fall over because a lightening bolt goes through my head when the sun hits my head. Then I have to wait a minute and then slowly open my eyes.

Other lighting, such as strobe lights, wreak havoc on Geneva's emotions, nervous system, and perception which may lead her to feeling nauseous, dizzy and provoke panic attacks.

Challenges with eye contact also emerged from the data. Matt explained, *“It is painful for me to look people in the eye … This lack of “eye contact” sometimes make people (especially teachers) think I'm not paying attention to them.*” Barbara explained that she avoided eye contact as well:

I can hear a person better if I don't look at their face. When somebody talks, I tend to turn my ear towards them, because I want to hear what they're saying…. Well, what I mean if I'm looking at them, it's kind of a mild distraction, because you know, if somebody is talking, I concentrate more on listening more than looking. So when I'm making an effort to listen, I'm not making an effort to look, so sometimes when I'm listening to somebody, I might look away from them, but I might turn my ear towards them.

At times, Barbara was able to make eye contact, yet it was atypical:

I feel that looking into someone's eyes is intrusive, like I'm staring at them. I have been criticized in the past for how I've looked at other people and about my facial expressions. I can do the right thing in the wrong way and not even know what it is that I'm doing wrong. If someone was doing something I was interested in, I might stare at them.

#### Touch

Participants described challenges with tactile input. Both hypo- and hyper- reactions to touch were described. Barbara described her hypersensitivity to fabric, sweat, and touch. She indicated that she does not wear any clothes that feel sticky or make her sweat. She only wears loose fitting clothes such as cotton or cotton polyester combinations. She also described having a sensitive scalp. Barbara recalled when she had long hair:

Hair was a big battle for me when I was growing up because you know how when your hair's long enough—it gets in the way, and even if you tied it back, the little fuzzies will work their way out and tickle your face. But I'm talking about if there were tangles in it, and I pulled it. That would drive me nuts… I over react to painful stimuli.

This reaction to painful stimuli was extremely problematic when it came to touching during medical procedures. As a child Barbara was very scared, over-sensitive and over-anxious about anything medical. She shared a story she had written about the experience.

Kathy-Xania explained her sense of touch as more hyposensitive. She said, *“I have a high pain tolerance, except around my mouth… I have a very high pain tolerance… I like deep pressure… I prefer deep pressure over light pressure.”* Like Barbara, Kathy-Xania also had an aversion to sweat. She stated:

I just don't like sweat. It's like disgusting. It's wet and sticky… But my neck—especially the back of my neck where my hairline is. Yeah, I just don't like it and I don't usually get as hot as easily as other people. But I don't like sweat. I think it's because I don't like wet feeling and sweat is wet.

Geneva explained that her sense of touch and pain is much different than others. She described that sometimes when she cut herself she most likely would not feel pain. She stated, *“I didn't feel the skin being pierced because I don't have normal feeling in my skin. I don't have normal sensitivity in my skin.”* She further explained how some of her body was unresponsive to some touch while other parts of her body (e.g., the back of her neck) were very sensitive. She said that in *“some areas my sensory system I have deficits, in other areas I have super sensitivity. That goes back and forth.”*

Geneva described avoiding touch from people she did not know well. She explained that if a person that, she had not seen touched her she would get *“scared out of my heebie jeebies—I will jump a foot in the air… Startled, heart pounding, panic attack.”* Geneva said that there were certain clothes that she was not able to wear mostly because of the material used in making the garment. For example, she needed to wear cotton underwear rather than synthetic underwear. If she did not wear the cotton underwear she would sweat, itch, and break out in a rash.

### Action

Participants revealed difficulties with controlling, executing, and combining movements. Most participants discussed difficulties controlling movements. Jean Paul described difficulty with holding his body still, particularly when he was nervous. Matthew spoke about difficulty controlling his actions, even basic day-to-day motor actions.

Barbara also discussed challenges with controlling her movements during times when she felt nervous, excited, or overloaded. She described, *“I had an automatic urge to touch my body—rub my thighs or my stomach and chest.”* Barbara expressed that she became upset and felt criticized when others did not understand her challenges related to controlling her actions:

I want to stop doing anything that doesn't look normal. But if I am feeling really bad inside, I want people to see the distress signals for what they are. I want people to understand I don't want to hide the urges if I'm feeling really bad. I want people to let me be. I've had all kinds of people who thought they were helping me stop doing things. I have been endlessly criticized about how different I looked, criticized about all kinds of tiny differences in my behavior. There's a point where you say to hell with it, its impossible to please you people…. No one ever tried to really understand what it was like to be me…. I wish they had accepted some of my behaviors I didn't have any control over. You don't criticize people with cerebral palsy.

Participants expressed challenges with execution of movement. Differences could result in problems with starting or stopping movements. Barbara discussed how she wished she had better coordination. Her difficulty and lack of coordination caused her frustration. Balance was also difficult for Barbara. Motor coordination was difficult for other participants.

Kathy-Xania had been told her movements were different. As she said, *“I was sitting on the floor and when I got up after looking at a couple of books, my friend said I got up like an animal does.”* She said that she was aware that her movements were different, but she was not quite sure how her movements differed from others. One observation she made was that her lack of depth perception had a dramatic affect on her movements. She said sometimes when she needed to go up the steps she got down on all fours. She said she was able to execute the movement of walking up the stairs on two feet, but it was very challenging. For that reason when she was at home or was unable to execute the movement she might need to *“crawl”* up the steps.

Participants mentioned challenges around combining two or more movements or actions. Geneva said that she was able to combine two tasks but she would easily *“lose the rhythm.”* She recalled the example of learning to dance:

I tried to learn a very simple line dance. I could not learn my footsteps and my hand movements at the same time. I had to teach my feet how to do it then stand still. I had to hold on to a rail, teach my feet their steps then lean against the wall with my feet out balancing me and learn my arm steps. Then hold on to the bar and learn my torso steps and then from there you learn what to do with the hips. Slowly, I turn the music on slow and I very, very, very slowly start the feet and very slowly add the hands then very, very slowly add the torso, etc. Everything has to be thought out, that is what is so annoying. There are just a very few things that I do two things at the same time without thinking them through as I am going.

At times, Geneva needed to separate tasks out while other times combining was necessary. For example, *“If I am running and I look away from the sidewalk, I'll trip on the next thing on the sidewalk.”*

### Postures

The trouble that some individuals with autism have with action may be due in part to differences in postures. A few experiences from participants as they related to posture are briefly noted.

As a teenager, Barbara was told that she grinned and that others *“… didn't like my posture or how I sat at the dinner table. My body just never seemed to be in a position that was acceptable.”* She explained that she did not choose her body postures rather than her postures were a result of the way her body positioned itself in space.

Other participants noted difficulties with proprioception and posture. Geneva, illustrated the difficulty in knowing where her body was in space. Geneva said not only was this *“a frustrating annoyance”* it could be life threatening as in the time *“where I almost died because I was drowning in a pool because I couldn't find up.”* Geneva said that she was best able to focus on the task at hand when she had some body awareness. For example she was better able to think and communicate during the interview because she was sitting with her body supported in a recliner or the bathtub. This accommodation of the recliner or bathtub assisted her both physically and emotionally.

### Emotions

Participants discussed challenges with expressing, controlling, identifying, and/or changing emotions. In addition, many participants spoke about specific accommodations that allowed them to manage their emotions more effectively.

Participants varied in how they described their challenges with emotions. Most felt they had the most trouble with expressing emotions. Barbara described challenges with both expressing and controlling emotions:

I think I've had times where I wasn't able to express how I was feeling and sometimes it was hard to experience my feelings directly. And one of the biggest problems was that I tried to express how I felt and people just didn't understand, my feelings were just so much different than another person that they just simply disregarded it.

She went on to say:

I had a problem with controlling my behavior. I did a lot of crying and a lot of complaining and I tried real hard to express my feelings to them, but people just didn't understand my feelings. I didn't have the same kind of feelings other people had.

Kathy-Xania described a different experience with expressing emotions. She stated:

I don't cry easily. I feel it inside, but I don't always show it on the outside. I think I get affected by things very easily… With me, it's all in my face. Usually if anything happens, emotionally, it's usually—my head gets affected first…. Expressing it and with me, expression tends to be hard… The emotions with people like me are much more intense. We have them, they're just intense and expressed differently… It's how we express it. I think it's there, but the expression is maybe different.

Participants discussed challenges with controlling and modulating their emotions. A few participants described their emotions as a *“roller coaster.”* Barbara described her emotional roller coaster connected by extreme depression and intense excitement: *“I could get very upset very easily, but I couldn't get over it.”* On the other hand, Barbara indicated, *“there have been lots of times that I wasn't really able to feel my feelings directly and there were some things I couldn't deal with directly because it was too painful.”* Kathy-Xania indicated that she, too, felt like she was on an emotional roller coaster. She explained:

There is a lot of rage in me and I think that is due to a lot of experiences that I have had. When I get rageful it is usually an event or an emotion or something that I have to be to work. Dealing with, like I am going to be going to the Social Security office on Tuesday with my social worker and just, well, I am trying to get health benefits and it just pisses me off to no end. If I read about the economy and read things like that—I start raging.

Challenges with identifying their own emotions were also discussed by participants. Geneva spoke extensively of this. She provided herself with a mental checklist that assisted her with identifying her own emotions. For example, she said:

I go into a room and I see somebody I knew in school. I don't fully remember the relationship because I didn't really know them that well. But an intense emotion comes inside of me… I have to stop and think are my hands sweating, is my stomach in a knot, is my face turning red or white, am I shaking or frozen in my steps, is my breathing fast or slow, do I feel a panic reaction or do I feel magnetically drawn. I have to go through this checklist until I get enough guesses to identify—Oh, I must have liked him.

Embarrassment would involve a different checklist. Geneva said, *“I wouldn't want to look but I did want to look. There would be a polarity between looking and not looking. My face would be warm and I would want to run in both directions….”* Panic *“poses a breathing off, makes us feel like we are in a straight jacket that is slowly being tightened….To us a panic attack is more like an attack of horror.”* The checklist for happy would be:

Somebody gives me something that I have never seen before, but I have never wanted one but it would be useful. I have to put it into a scenario to make it make sense. When I discover my hands are trembling ever so slightly and I have got this giggle inside my stomach and when I look at my face on the inside I have got this smile, this itty bitty smile, and I am looking around at other people, especially the gigglers, and I try to pick up on what they might be thinking or what they are saying and I would go through this and the last thing that would go make up my mind would be do I want to put it under the table or do I want to take it home? Do I want to accidentally leave it under the table or take it home?

Participants also talked about difficulty identifying or understanding the emotions of other people. Kathy-Xania explained that she could identify and understand anger, friendship, loyalty, and dishonesty. She expressed that she has difficulty understanding sexuality and jealousy. As she explained:

I mean all the sexuality stuff I have really very little understanding of. I acknowledge it. I know it exists, the emotions people have about that area—I just don't understand. I have envy and jealousy myself but not over things of other people…. It's hard for me understand why they would have those feelings.

Although Kathy-Xania and other participants stated that they have difficulty identifying the emotions of others, all participants disagreed with the assumption that individuals with autism lack a theory of mind or are unable to take the perspective of others. Participants expressed feelings they experienced and also spoke about relating to another person's feelings. It was apparent that for these participants there was a difference in understanding emotions, not an absence.

Participants talked extensively about accommodations they have used to manage their emotions. Barbara described often hiding her emotions and isolating herself, however, this made her feel miserable. She explained that when she personified objects and projected her feeling to that object, she felt better:

It seemed in order for me to have any happiness, I had to personify objects and treat them like they were human….Well, lots of times I would project my feelings onto something, rather than being able to feel them directly…. I don't know how to explain it. Lots of times I would say the feelings that I had I wasn't able to feel was like maybe feelings of pleasure—like for me to enjoy something—I'd have to sense that one of my fantasy objects enjoyed it too.

Barbara further explained:

When I could interact with a personified object I felt content. For example, I personified the building where I went to high school and called it Troy. When I could talk to the building or interact with it, I felt content. At that time I got no good feelings from being around people. There was always tension when I was around people because I never fit in and I had nothing in common with people and there was no sense of connection, objects were my only source of comfort.

Two other participants also described personifying objects. Geneva personified a large stuffed rabbit. This personification provided Geneva with a comforting feeling. Kathy-Xania found the feeling of security when holding wooden puzzle pieces of California, Texas, Montana, and Africa.

Participants talked about the impact of stress on both their emotions and their behavior. Barbara explained that she tried to *“stay out of a situation where I am stressed. Otherwise, there is no controlling my emotions.”* She described feeling stress, nervousness, and depression caused by criticism from others regarding her behavior. Barbara added:

The more people that criticized me—what it did was made me angry and want to rebel. When people criticized me a lot I just didn't like being around them and I got angry a lot and I cried a lot. And it just simply—it caused more tension. In fact it just took a bad situation and made it worse. There were times when I was in a situation where I sometimes had to act a little bit silly to keep myself from getting upset because if I would have had that defense mechanism I would have just fallen apart…. Well sometimes I felt so nervous that sometimes I would have to act silly. In order to keep from getting upset…. If I got upset I stayed that way. I had to really do everything I could to try and avoid getting that way….because I have been hurt so much. I have so much bitterness. And I have to deal with forgiving a lot of people.

Barbara further explained that, she felt isolated and that no one understood how hard things were for her; instead they only focused on her outward behavior. As she stated, *“No one cared about how terrible I felt from the inside.”* Barbara wanted others to understand, help her understand, and to support her. Jean Paul agreed. He said that connecting with and providing feedback to a person with autism on his/her level without criticism was extremely helpful.

Matt described a variety of strategies to deal with stress. He explained that some books, pictures, and electronic equipment (e.g., computers, Game Boy) reminded him of home. This memory of a quiet, safe place created a calming feeling and allowed him to better deal with stress. While these and other strategies were helpful, he explained that other people often did not understand his strategies. He explained others' reactions to his strategies:

Some of these things upset my teachers because they don't understand why I do them. And I couldn't communicate well enough to explain things, even to myself. For example, my parents said I banged my head a lot when I got frustrated when I was young. But I usually banged it on soft things so it didn't hurt much. Sometimes when I am mad now I still swing my head through the air. But I don't hit anything with my head. Head banging motions help me deal with my nervousness.

### Communication

Participants described challenges with both verbal and nonverbal communication. Specifically participants described challenges with speech execution and control, rhythm in conversation, and using and understanding nonverbal communication. Many described the dynamic interaction between speech and emotions.

Barbara described difficulty with speech execution and control. She described how emotional reactions caused changes in her ability to control her speech:

I know my voice is loud now—but when I talk about emotional things, it just bursts out of me—there's just so much pressure. In fact, lots of times my voice sounds bad—it's only part of the emotion—you know—it's sort of like be thankful it's only a whine—I'm holding in a scream…. But if an autistic person's voice is loud, it's not because they're trying to be loud, it's because there's tension there. An autistic person is sitting on a powder keg of emotions. And it's gotta go somewhere—and perhaps talking loud is the only way they can get any relief from that tension. It's the only outlet that I had that worked. If I was angry, I could exercise, I could do anything but it wouldn't do any good. Talking or yelling or something was the only thing that gave me any relief at all…. My voice was the only emotional release. It was the only safety valve on that pressure. But the funny thing about it—was the more people nagged me, the more it aggravated what it was they were nagging me about.

Kathy-Xania also described difficulty controlling her speech and vocal outbursts when she was emotional, even if the emotion was excitement. She described often making *“uncontrollable sounds”* when she heard the name of a geographic location.

Kathy-Xania also commented on how difficult it was for her to understand the rhythm or pattern in conversations. She explained that this was exacerbated when the subject matter was *“historical or talking about something I really like. I want to jump in there you know. But I never know when to jump in and often get it wrong.”*

Participants also described challenges with becoming stuck in words or phrases and/or sounds. Jean Paul described repeating words and phrases over and over again. As he said, *“I could not stop, even when I wanted to.”* Geneva described similar challenges with speech execution:

There would be a lot of times that I would stop in mid-word and maybe repeat a syllable and go into verbal perseverations. I would start to say things, I would use the wrong words. Like I would say: ‘let's go into that store’ in my mind, but the words would come out ‘let's go in that box’…. I would lose my train of thought constantly.

Participants also described challenges with using and understanding nonverbal communication. Barbara said, *“As a child growing up, as an autistic person, I could not read body language…. because all that was too abstract.”* She further explained:

I don't understand body language. And I had very little body language. My face tended to have a blank expression on it a lot. And I did not have body language or understand body language but they put all the burden of that on me. As if I was suppose to change it. It was neurological but they didn't recognize anything as neurological. I think eventually I started to develop more body language. But it was just something that took a long time to develop. But one of the things that happened is that I had a very high level of nervous tension so I just looked and acted very nervous. And a lot of times I grinned a lot because of tension. And lots of times I laughed so I wouldn't cry because I knew if I didn't act silly that I would get upset and I really had to struggle just to keep myself together.

Barbara further explained:

I'd sometimes look like I wasn't paying attention because I'd be preoccupied or I'd grin, or I'd grimace, or I'd frown. I got criticized for my facial expressions. I got criticized for things that happened automatically. I got criticized for things I had no control over. Things other people don't think about. Normal people's faces look like they're supposed to look—when you're autistic—your face does not look like you're supposed to look. Different things go on inside you. Different things show on the outside. It's automatic. Nobody sits around and says I'll move this muscle here—I'll move this muscle there—I'll put this muscle there—they don't stare in a mirror and think—move this muscle, move that muscle, yeah that's the look and practice that. Nobody does that. But my facial expressions I got criticized for.

### Cognition

Data from the categories “memory” and “thought” were combined into the category of “cognition.” Thought processes of the individuals from this study proved unique and distinctive.

Geneva had been told that she was born mentally gifted. She described her IQ scores:

What they didn't know was that I may have been as high as 150 to begin with. I am about 135 now, but I may have been as high as 150, but I used everything over 100 to pass in society. So I brought it down, down, down to where now it is 135 where it should be 150 but I have to pass in society.

She described that *“passing in society or keeping your outward appearance looking typical”* required a *“huge portion”* of her mental energy. As a result she created a variety of accommodations to reduce the amount of energy or thinking needed to complete a cognitive task. She summarized some of her thinking and the accommodations she had created for herself to be a more effective thinker. For example, Geneva described her optimal studying experience in college:

I would go in the bathroom and start the bath water, right? Then I would get this desk I had made to go across the bathtub, then I would put the notebooks on there and my textbook and then I would put the tape recorder with the taped book from books for the blind on the commode seat and I would read the text, hear the tape and take notes at the same time and if at any point something happened to my concentration that it stopped or something, I could just stop the tape and go back.

This atmosphere seemed to organize her system. Geneva explained further using a computer analogy:

Now, comparing my mind to a computer, it is like I have the input card but the output card is all jumbled up. There is no alphabetical order and half of them are missing but the input is there. But I can't get to the output…. I have no idea why. Somebody will ask me a question and I will say I will have to get back to you and I just have to forget about it and walk around till the stuff pops up and it pops up eventually or sometimes it doesn't.

Geneva also described that one of the major difficulties she experienced was because:

People don't realize the major problem that nobody ever sees or realizes is how much conscious thinking we have to do just to function. Walking takes thinking. So if I am walking and you ask me a question I could trip or I could mess up the sentence and put the wrong word in. Or have to stop and say, ‘what did you say?’ I can walk with my girl friend down the street and carry on a conversation as long as she is right there but I have to look down at the sidewalk. I have to keep track of where the sidewalk is and where any obstacles are and all that stuff and sometimes if I have to keep walking and I feel like I am going to blow any second I make sure the path is clear ahead of me and close my eyes and continue walking.

Kathy-Xania described herself as an *“entirely visual thinker.”* She said that the way she thought was:

Similar to Temple Grandin—and that's why I like countries and states so much. Because it is all visual to me. History is movement. It is movement. Economy moves and countries move. People, countries and their governments have their ups and downs and I like looking at pictures and maps and flags. It is all visual to me. It is like a story. I can just visualize it all.

Kathy-Xania's mind also connected many ideas and words. For example, she enjoyed hearing where people were from. When she heard one of the researchers was from Wisconsin she said that the first thoughts that came to her mind were *“University of Wisconsin, Madison, Cheese, History, my name is Yon Yonson and I come from Wisconsin.”* In fact, she voiced some of these thoughts during the interview. She said:

I always have to know what city people come from but there are times I don't always ask, especially if I am dealing with a business situation or whatever. I usually deal with business at hand. But usually I am very compelled to know what city people are from or places they have been to, you know.

Barbara's thought process also involved such connections. These connections however, often lead to intrusive thoughts. She stated:

I think in some situations it's just harder for me not to have intrusive thoughts. Some autistic people, they say, block things out or they shut things down or whatever. My mind doesn't think—I'm not able to stop an intrusive thought or block something out unless it's something really, really, really mild—but if it's severe, it all comes in and there's no way I can stop it. I'm not able to tune out anything…. Intrusive thoughts would be nonsense syllables or something. I don't understand why this is—but if I was trying to study a foreign language or if I tried to study anything with odd-sounding words, I'd get nonsense syllables and stuff would pop in my mind and anxiety. It doesn't make a bit of sense. I don't know why it happens.

As a result, *“it takes a lot of concentration and I'm not able to process that much information at one time.”* Barbara stated that when she does not put forth a great deal of conscious effort she has a hard time staying focused. She explained:

Like if I was in the music room and I saw a musical instrument or the record player was turned off, I would have intrusive thoughts about songs in my mind. Or if I was trying to read my geography assignment, a whole bunch of nonsense syllables would pop into my mind and would be triggered by funny-sounding names. Just stupid things like that—that wouldn't amount to a hill of beans, but I would just get this terrible anxiety and boy, I would just scream.

Matt explained that, excessive stress could be problematic for his processing of thoughts. He described how some people pressure him by yelling at him to respond. He explained, *“This type of pressure causes my thought processes to ‘crash’ like an overworked computer disk. It's like my thoughts are trying to get out of my head at once and I can't deal with it.”*

Barbara and Kathy-Xania each explained that sometimes they had *“cognitive overload.”* This might happen when either of them had difficulty integrating different areas such as thought, perception and action. Both gave the example of driving. Barbara explained her experience of driving:

When you're driving you have about 20 different things you have to keep track of—traffic going in all different directions—you have to watch the traffic, the light's red, then it's green, then it's red, then it's green—you have to pay attention to whether the light's red and green and go to the corner—pay attention to the speed limit and the signs and staying in your lane and watching all the other cars at the same time—I could pay attention and not see something else. I might avoid hitting a car only to hit another one.

## Discussion

We started this project with the conceptual model of sensory and movement differences and a conviction that it is important to listen to people with autism. This model was based on that offered by Hill and Leary (1993), Donnellan and Leary (1995), Leary and Hill (1996), and Leary and Donnellan (2012). Sensory and movement differences is a disruption in the organization and regulation of perception, action, posture, language, speech, thought, emotion, and/or memory. This definition guided data collection and analysis. We found that the data strongly supported the presence of disruption of organization and regulation of sensory and movement differences in the lived experience of these participants with autism. Typically developing people experience sensory and movement differences as well, of course. However, the present data suggests that in autism this disruption of organization and regulation is amplified in terms of quantity, quality, intensity, and may affect everyday life. For example, recall how Barbara found it almost impossible to let go of an intrusive thought, how she was often not able to move past a negative emotion, or how the effects of an unpleasant noise lasted much longer than the noise itself.

The professional literature on autism, from Kanner (1943) to the present relies heavily on the “etic” or outsider view (Pike, 1950; Berreman, 1966). The assumption, unstated but generally operationalized, is that our experience of these individuals is essentially the same as their own. Here, we have attempted to offer the “emic” view, i.e., at least some of their experience, in their own voices. With this information, we might begin to understand that when Barbara looks away in a conversation it could be her best accommodation in order to understand our words. And perhaps we could see her behavior as less a social inadequacy that fits our definition of autism than an individual's best attempt to overcome a sensory problem that otherwise would interfere with her attempt to interact. And, this information might inform teachers and therapists who have been taught that they must get eye contact before providing instruction. In another example, recall that Geneva described her need to think about how to walk in order to walk. For most non-autistic individuals, this is automatic, smooth, and fluid. She described further challenges when combining walking and speech. Geneva explained that she had to look down at the sidewalk while having a conversation with a friend so that she could continue to talk and walk. Without knowing this was an accommodation used by Geneva one might assume her behavior indicated a social deficit.

Additionally, these data suggest that sensory and movement differences are not the same for all people with the autism label, nor always the same for any given person. Moreover, those of us who support people with autism should be mindful that:

Movement disturbance can clearly have a profound effect on a person's ability to regulate movement in order to effectively communicate, relate, and participate with others. Once this possibility is acknowledged, it becomes necessary to suspend absolute trust in one's intuitive interpretation of actions and intent. Behaviors may not be what they seem. (Leary and Hill, 1996, p.44)

Our understanding of each individual requires awareness of the dynamic, multi-layered, contextually determined aspects of organization and regulation. It is a tall order, worthy of our attention.

For the purpose of this study, it was necessary to describe various areas separately, it is important to remember that perception, action, emotion, communication, cognition, and posture operates in an interactive dynamic fashion (see Thelen and Smith, 1994; Thelen, 1995). Continual interaction occurs across the areas in a dynamic process. These connections seem to be dependent on context. Context includes a wide variety of factors not limited to overt, observable stimuli. Context also includes emotional status, environmental stimuli, memory triggers, etc. Recall that many participants discussed the effect of stress on their ability to organize and regulate their perceptions and movements. In other words, a person's ability to function is highly dependent on context, which is ever shifting, and the unique and intimate interconnections of the various areas may contribute to sensory and movement difference for an individual with autism at any moment in time.

One obvious implication is that “interventions,” medical, behavioral or educational, ought not be pre-packaged nor assumed to work for all people with autism. They must be personalized accommodations (Luria, 1932/1976; Sacks, 1990) and personalization requires that we “learn to listen” (Lovett, 1996) to the individual rather than rely on our preconceived notions of our own expertise on the topic. Moreover, it must be said that each of the participants (and many others with the autism label to whom we have spoken) expressed gratitude for the information about sensory and movement differences. While they knew their own experience, and could talk about it, they did not know that others had similar experiences. Moreover, they thought they were to “blame” for their challenges, because they had so often been blamed by others and subjected to so much behavioral modification with the goal of eliminating behavior beyond their own volition. The separation of mind from body noted earlier (Damasio, 1994) has contributed to this situation where the literature concentrates on “mind,” leaving most autistic people to deal with problems of their bodies on their own. Their experience described here and in first-hand accounts suggests that a change on our part as professionals is essential. With sensitivity and humility about how little we actually know compared to what we think we know; this more personalized path could have significant effect in some lives affected by autism (Donnellan, 1999).

We are not saying sensory and movement differences are the cause of autism; in fact, occasional challenges or differences in perception, action, emotion, communication, cognition, posture are part of our shared human experience. We all occasionally forget why we went into a particular room and have to return to the original context to remember. We sometimes have trouble with a sound or a touch under the “wrong” circumstances, for example. For people with the autism label, however, these differences may have at least the following effects: (1) that sensory and movement differences may be more problematic for people with autism because of the magnitude of differences they experience with intensity, duration, rhythm, rate, frequency and /or timing of movement they experience; (2) events, stimuli and experiences in the world seem to elicit different response in some people with autism than the typical patterned response of other non-labeled people; and (3) areas may affect these individuals in an unusual and dynamic fashion which is highly dependent upon external and internal context.

Interviewing individuals with autism about sensory and movement differences was challenging. At times, despite their interest in the topic, it was difficult to know the “right” questions to elicit information in some areas, such as posture and cognition. Though the vignettes helped, it was not always clear if the participant actually experienced differences but was unable to articulate the information or if the person did not experience challenges in a particular area. Each of the categories discussed in this study warrant further investigation.

These individuals are considered “high functioning,” and yet live with challenges that are poorly understood by their community, colleagues and peers and seldom reflected in professional descriptions and studies. Despite the occasional difficulties, qualitative research that seeks the perspectives and experiences of people with autism is essential if we seek to understand how sensory and movement differences impact these individuals. In particular, we need to explore whether and to what extent these sensory movement difficulties create or contribute to the difficulties that we experience as impairments in social interaction, communication and behavior. We must listen carefully to individuals with autism and be willing to incorporate their perspectives into our learning.

Many experts in the field of autism, especially and specifically people with autism (Kathy-Xania, interview; Barbara, interview; Geneva, interview; Ne'eman and Kapp, personal communication, July 29, 2012), disagree with much of the explanation of autism currently available in the autism literature. Kathy-Xania (interview) and others (Mackay, 2003; Biklen, 2005) suggest that there be more qualitative studies to gain the perspective of people with autism. This study supports the notion that more qualitative research, including in-depth interviews, case studies and first-hand accounts, that elicit the experiences and perspectives of individuals with autism would be prudent. These data would contribute to a more expansive view that incorporates the possibility that autism is a disorder that affects motor planning, behavior, communication, the sensory motor system, and the dynamic interaction of all of these (Herbert, 2012). Current definitions may fail to communicate the depth, breadth, and infinite variability in the experience of autism.

Five participants identified with the label of autism provided data for this study. People with autism have well documented social and communication difficulties. For that reason, a variety of methods were used to obtain meaningful data.

For in-depth interviews, we selected only verbal people with autism who were able to articulate at least a portion of their experiences. All research participants were able to communicate independently. This may have limited the generalizability to individuals with autism who are not able to articulate in the conventional manner or through augmentative or alternative forms of communication. Furthermore, all the participants in the study were white middle to upper-middle class. In addition, the ratio of females to males in this study (3:2) is not representative of the ratio of females to males in the literature, which is one to four/five. Certainly further inquiry is needed that explores the experiences of less articulate verbal and nonverbal men and women with autism as well as exploring the experiences of individuals with autism from various socioeconomic backgrounds. There is sufficient information in the first-hand accounts of autism to suggest that such inquiry would be a contribution to our understanding of all people with the autism label (Barron and Barron, 1992; Grandin, 1992, 1995; Williams, 1992, 1994; McKean, 1994; Blackman, 1999; Hale and Hale, 1999; Mukhopadhyay, 2000; Biklen, 2005; Goddard and Goddard, 2012).

Finally, this research raises new questions that, when answered, may further expand current definitions and understanding of autism. How do differences in magnitude of intensity, duration, rhythm, rate, frequency and /or timing of movement create challenges for people with autism? Do differences in intensity, duration, rhythm, rate, frequency and /or timing of movement occur more frequently in people with autism? Why do different type of events, stimuli and experiences in the world elicit different responses in some people with autism? For example, how does pain tolerance and internal auto-regulation of temperature impact individuals with autism? What is it about the areas of perception, action, posture, emotion, communication, and cognition that affect these individuals in an unusual and interdependent fashion? What is the role of external and internal context on the experiences of individuals with autism? How might current treatments and teaching strategies be modified to include the possibility of sensory and movement difference in autism? What types of personalized accommodations are helpful to individuals with autism?

We hope this research will serve as a catalyst for additional studies that explore the experience of sensory and movement differences in autism. This will encourage the collaboration of individuals with autism and professionals in fields such as neurology, psychiatry, neuroscience, education, psychology as well as basic biological sciences so that autism is explored through the lens of more recent relevant research on how the brain and body work.

### Conflict of interest statement

The authors declare that the research was conducted in the absence of any commercial or financial relationships that could be construed as a potential conflict of interest.

## Footnotes

^1^For additional references of sensory and movement literature see Leary and Hill, 2012, pp.101–115

## References

[B1] American Psychiatric Association. (2000). Diagnostic and Statistical Manual of Mental Disorders, 4th Edn., text revision. Washington, DC: Author

[B2] Barron J. Barron S. (1992). There's a Boy in Here. New York, NY: Simon and Schuster

[B3] Berreman G. (1966). Anemic and emetic analyses in social anthropology. Am. Anthropol. 68, 346–354

[B4] Biklen D. (2005). Autism and the Myth of the Person Alone. New York, NY: New York University Press

[B5] Blackman L. (1999). Lucy's Story: Autism and Other Adventures. Brisbane, QLD: Book in Hand

[B6] Bleuler E. (1911/1950). Dementia Praecox or the Group of Schizophrenias. Trans. Zinkin J. New York, NY: International Universities Press

[B7] Broderick A. Kasa-Hendrickson C. (2001). “Say just one word at first”: the emergence of reliable speech in a student labeled with autism. Res. Pract. Pers. Sev. Disabil. 26, 13–24

[B8] Cesaroni L. (1990). Exploring the Autistic Experience Through First-Hand Accounts from High-Functioning Individuals with Autism. Unpublished document, University of Toronto

[B9] Charmaz K. (2000). Grounded theory: objectivist and constructivist methods, in Handbook of Qualitative Research, 2nd Edn, eds Denzin N. Lincoln Y. (Thousand Oaks, CA: Sage Publications), 509–535

[B10] Charmaz K. (2006). Constructing Grounded Theory: A Practical Guide Through Qualitative Analysis. London: Sage Publications 10.1093/geront/gns046

[B11] Damasio A. R. (1994). Descartes Error: Emotion, Reason, and the Human Brain. New York, NY: G.P. Putnam

[B12] Donnellan A. M. Leary M. R. (1995). Movement Differences and Diversity in Autism/Mental Retardation. Madison, WI: DRI Press

[B13] Donnellan A. M. Leary M. R. Robledo J. (2006). I can't get started: stress and the role of movement differences for individuals with the autism label, in, Stress and Coping in Autism, eds Baron G. Groden J. Groden G. Lipsitt L. (Oxford: Oxford University Press), 205–245

[B14] Donnellan A. M. (1999). Invented knowledge and autism: highlighting our strengths and expanding the conversation, JASH 24, 230–236

[B15] Ferguson P. Ferguson D. Taylor S. (1992). Interpreting Disability: A Qualitative Reader. New York, NY: Teachers College Press

[B16] Goddard P. Goddard D. (2012). I Am Intelligent: From Heartbreak to Healing – A Mother and Daughter's Journey Through Autism. Guiford, CT: Globe Pequot Press

[B17] Grandin T. (1992). An inside view of autism, in High-Functioning Individuals with Autism, eds Schopler E. Mesibov G. B. (New York, NY: Plenum), 105–126

[B18] Grandin T. (1995). Thinking in Pictures and Other Reports from My Life with Autism. New York, NY: Doubleday

[B19] Hale M. Hale C. (1999). I Had No Means to Shout! Bloomington, IN: 1st Books

[B20] Herbert M. (2012). The Autism Revolution: Whole-Body Strategies. New York, NY: Ballantine Books

[B21] Hill D. Leary M. (1993). Movement Disturbance: A Clue to Hidden Competencies in Persons Diagnosed with Autism and Other Developmental Disabilities. Madison, WI: DRI Press

[B22] Jolliffe T. Lansdown R. Robinson C. (1992). Autism: a personal account. Communication 26, 12–19

[B23] Kahlbaum K. (1874/1973). Catatonia. Trans. Levij Y. Pridan T. Baltimore, MD: Johns Hopkins University Press

[B24] Kanner L. (1943). Autistic disturbances of affective contact. Nerv. Child 2, 217–250 4880460

[B25] Leary M. R. Donnellan A. M. (2012). Autism: Sensory-Movement Differences and Diversity. Cambridge, WI: Cambridge Book Review Press

[B26] Leary M. R. Hill D. (1996). Moving on: autism and movement disturbance. Ment. Retard. 34, 39–53 8822025

[B27] Leary M. R. Hill D. Donnellan (1999). Autism: Myths and Misunderstandings. Presented to the School of Psychology, University of Michigan, Ann Arbor

[B28] Lincoln Y. S. Guba E. G. (1985). Naturalistic Inquiry. Beverly Hills, CA: Sage

[B29] Lovett H. (1996). Learning to Listen: Positive Approaches and People with Difficult Behavior. Baltimore, MD: Paul, H. Brookes

[B30] Luria A. R. (1932/1976). The Nature of Human Conflicts: Or Emotion, Conflict and Will. New York, NY: Liveright

[B31] Mackay R. (2003). “Tell them who I was”: the social construction of aphasia. Disabil. Soc. 16, 811–826

[B32] McKean T. A. (1994). Soon Will Come the Light. Arlington, TX: Future Education

[B33] Mukhopadhyay T. R. (2000). Beyond the Silence. London: National Autistic Society

[B34] Patton M. Q. (2002). Qualitative Research and Evaluation Methods, 3rd Edn Thousand Oaks, CA: Sage 10.1016/j.nepr.2005.05.001

[B35] Pike K. L. (1950). Axioms and Procedures for Reconstructions in Comparative Linguistics: An Experimental Syllabus. Glendale, CA: Summer Institute of Linguistics

[B36] Robledo J. Donnellan A. M. (2008). Properties of supportive relationships from the perspective of academically successful individuals with autism. Intellect. Dev. Disabil. 46, 299–310 10.1352/1934-9556(2008)46[299:POSRFT]2.0.CO;2 18671444

[B37] Rogers D. (1990). Psychiatric consequences of basal ganglia disease. Semin. Neurol. 10, 262–266 10.1055/s-2008-1041277 2259804

[B38] Sacks O. (1990). Awakenings, 6th Edn New York, NY: Harper Perennial

[B39] Strandt-Conroy K. (1999). Exploring Movement Differences in Autism Through First-Hand Accounts. Doctoral Dissertation, University of Wisconsin, Madison

[B40] Strauss A. L. (1987). Qualitative Analysis for Social Scientists. Cambridge, UK: Cambridge University Press

[B41] Thelen E. Smith L. B. (1994). A Dynamic Systems Approach to Development and Cognition. London: MIT Press

[B42] Thelen E. (1995). Motor development: a new synthesis. Am. Psychol. 50, 79–95 7879990 10.1037//0003-066x.50.2.79

[B43] Volkmar F. R. Cohen D. J. (1985). The experience of infantile autism: a first-person account by Tony, W. J. Autism Dev. Disord. 15, 47–54 3980429 10.1007/BF01837898

[B44] White G. B. White M. S. (1987). Autism from the inside. Med. Hypothesis 24, 223–229 10.1016/0306-9877(87)90068-5 3696026

[B45] Williams D. (1992). Nobody Nowhere. London: Doubleday

[B46] Williams D. (1994). Somebody Somewhere. New York, NY: Times Books

